# Load-induced blood marker kinetics in patients with medial knee compartment osteoarthritis are associated with accumulated load and patient reported outcome measures

**DOI:** 10.12688/f1000research.131702.1

**Published:** 2023-03-17

**Authors:** Annegret Mündermann, Corina Nüesch, Simon Herger, Anna-Maria Liphardt, Frédérique Chammartin, Enrico De Pieri, Christian Egloff

**Affiliations:** 1 Department of Orthopaedics and Trauamtology, University Hospital Basel, Basel, 4031, Switzerland; 2 Department of Biomedical Engineering, University of Basel, Allschwil, 4123, Switzerland; 3 Department of Clinical Research, University of Basel, Basel, 4031, Switzerland; 4 Department of Spine Surgery, University Hospital Basel, Basel, 4031, Switzerland; 5 Department of Internal Medicine 3 - Rheumatology and Immunology, Universitätsklinikum Erlangen & Friedrich-Alexander-Universität (FAU) Erlangen-Nürnberg, Erlangen, 91054, Germany; 6 Department of Internal Medicine 3 - Rheumatology and Immunology, Friedrich-Alexander-University Erlangen-Nuremberg (FAU), Erlangen, 91054, Germany; 7 Deutsches Zentrum Immuntherapie, Universitätsklinikum Erlangen & FAU Erlangen-Nürnberg, Erlangen, 91054, Germany

**Keywords:** Knee osteoarthritis, articular cartilage, serum biomarkers, stress test, in vivo mechanoresponse, ambulatory load, PROMs

## Abstract

**Background:** This study aimed to quantify the mechanoresponse of 10 blood marker candidates for joint metabolism to a walking stress test in patients with knee osteoarthritis and to determine the association among marker kinetics and with accumulated load and patient reported outcomes.

**Methods:** 24 patients with knee osteoarthritis completed questionnaires, and a walking stress test with six blood serum samples and gait analysis. Concentrations of cartilage oligomeric matrix protein (COMP), matrix metalloproteinases (MMP)-1, -3, and -9, epitope resulting from cleavage of type II collagen by collagenases (C2C), type II procollagen (CPII), interleukin (IL)-6, proteoglycan (PRG)-4, A disintegrin and metalloproteinase with thrombospondin motifs (ADAMTS)-4, and resistin were determined by enzyme-linked immunosorbent assays, Joint load (moments and compartmental forces) was estimated using musculoskeletal modeling using gait analysis data.

**Results:** COMP and MMP-3 showed an immediate increase after the walking stress followed by a decrease. MMP-9 and resistin showed a delayed decrease below pre-stress levels. ∆COMP correlated with ∆MMP-3 for most time points. ∆MMP-9 correlated with ∆resistin for most time points. The load-induced increase in blood marker levels correlated among blood markers and time points. C2C and resistin correlated positively and C2C/CPII and MMP2 correlated negatively with load during gait. Immediate relative ∆CPII and ∆MMP1 and delayed relative ∆COMP, ∆IL6, ∆C2C, ∆CPII, ∆MMP1 and ∆MMP3 correlated with the load accumulated during the walking stress. Baseline C2C levels correlated with Knee Osteoarthritis Outcome Score (KOOS) subscales and load-induced changes in MMP-3 with KOOS and Short Form 36 quality of life subscores (P<0.05).

**Conclusions:** The distinct and differentiated physiological response to the walking stress depends on accumulated load and appears relevant for patient reported osteoarthritis outcome and quality of life and warrants further investigation in the context of disease progression.

ClinicalTrials.gov registration: NCT02622204

## Introduction

The response of biomarkers of tissue biology to mechanical stimuli in physiological and pathological conditions has attracted increasing attention in recent years. ^1^ Introducing a walking stress test involving a 30-minute walking exercise, Mündermann *et al.* ^2^ reported consistent immediate effects of the walking stress on cartilage oligomeric matrix protein (COMP) with a return to pre-stress levels within 30-minutes after the exercise in young healthy adults. This mechanoresponse was clearly attributed to the mechanical stimulus confirmed by a control experiment without a stimulus. Patients with medial compartment knee osteoarthritis (OA) also showed an immediate increase in COMP but continuously decreasing levels below pre-stress levels after the walking stress without differences to an age-matched asymptomatic control group. ^3^ The change in COMP at 3.5 and 5.5 hours, ^4^ in a collagen degradation marker C1,2C and in the synthesis marker CS846 5.5 hours after the stimulus ^5^ predicted changes in articular cartilage thickness supporting the potential relevance of load-induced changes in blood markers in the progression of knee OA.

To date, most studies only investigated changes in COMP and reported an increase in COMP levels during a 20 to 45 minute walking exercise or up to 5000 steps ^2^^–^^4^^,^^6^^–^^15^ with a return to pre-stress levels within 30 minutes ^2^^,^^3^^,^^7^^,^^9^^,^^11^ or 60 minutes after the exercise. ^10^ Although load-induced marker kinetics may be affected by OA and differ in magnitude and timing between markers, ^3^^–^^5^ only few studies have reported on the kinetics of other markers to walking, marker kinetics beyond 60 minutes after the exercise or other mechanical stimuli in patients with OA. ^1^ Interestingly, Jayabalan *et al.* ^14^ reported an increase in COMP but not in tumor-necrosis-factor-α (TNF-α) after a 30-minute walk in patients with unilateral knee OA, and hence not all blood marker candidates may respond to mechanical stimuli in patients with knee OA. Correlations among blood marker kinetics may reveal sets of biomarkers involved in the same physiological pathways.

Candidates for mechanosensitive blood markers of joint pathology include markers of cartilage synthesis, cartilage degradation, joint lubrication or inflammation including the C-propeptide of type II procollagen (CPII), ^16^ the C2C epitope cleavage of type II collagen, ^17^^,^^18^ COMP, ^19^ matrix metalloproteinase (MMP)-1 ^20^ or MMP-3. ^21^ A disintegrin and metalloproteinase with thrombospondin motifs (ADAMTS-4), ^22^^,^^23^ proteoglycan 4 (PRG-4) or lubricin, ^24^ interleukin 6 (IL-6), ^25^^,^^26^ and resistin. ^27^^–^^30^

Baseline patient reported osteoarthritis outcome and quality of life (QoL) may predict future changes in blood markers of articular cartilage as suggested in a study on volleyball players, ^31^ and selected blood and urinary markers appear to be related to radiographic evidence of joint damage and/or clinical symptoms of OA. ^32^ To date, it is unknown whether the response of blood markers to load is associated with patient reported osteoarthritis outcome and QoL in patients with knee OA. Moreover, as none of the previous studies estimated the load accumulated during the respective exercise stress, to date the association between accumulated load and load-induced marker kinetics is unknown. Our exploratory study aimed (i) to quantify the mechanoresponse of this array of potential blood markers for joint pathology (COMP, MMP-1, MMP-3, MMP-9, CPII, C2C, C2C/CPII, ADAMTS-4, PRG-4, IL-6 and resistin) to a walking stress test in patients with knee OA and to determine the correlation (ii) among the kinetics of these blood markers, (iii) with accumulated knee load during the walking stress, and (iv) with patient reported osteoarthritis outcome and QoL.

## Methods

Data presented in this exploratory cross-sectional laboratory study were collected as part of the baseline assessment of a larger study on the effect of high tibial osteotomy on load-induced changes in cartilage biomarkers. ^33^ Participants were recruited from the Department of Orthopaedics and Traumatology at the University Hospital Basel. Inclusion criteria were radiographically diagnosed and isolated symptomatic medial compartment knee OA and being scheduled for high tibial osteotomy. Exclusion criteria were: use of walking aids; inability to walk for 30 minutes; age < 18 years (before maturation) or age > 70 years (risk of advanced general sarcopenia (degenerative loss of muscle mass in aging)); body mass index (BMI) > 35 kg/m ^2^; active rheumatic disorder; prior neuromuscular impairment (e.g. stroke); conditions other than knee OA that could cause abnormal patterns of locomotion; prior hip, knee, and ankle prosthesis or osteotomy of the lower extremities; prior spine surgery; other major medical problems; and current enrollment in another experimental (interventional) protocol. The study was approved by the regional ethics board (August 19, 2015; Ethikkommission Nordwest- und Zentralschweiz EKNZ 2015-224) and conducted in accordance with the Declaration of Helsinki, and participants provided written informed consent prior to participation.

### Participants

24 patients (16 male, 8 female) with medial compartment knee OA met the inclusion criteria and participated in this study. Patient demographics, disease characteristics (Kellgren Lawrence (KL) ^34^ grade) and the patient reported outcomes measures (PROMs, patients reported function: Knee Injury and Osteoarthritis Outcome Score (KOOS) ^35^; patient reported quality of life: 36-Item Short Form Health Survey (SF36) ^36^; KOOS and SF36 assessed immediately prior to the walking stress test) are shown in Table 1. We considered all KOOS subscores but used only the total SF36 score and its subscores physical function and pain for further analysis.

**Table 1.** Patient characteristics.

Parameter	Statistics (N=24)
*Demographics (mean ± 1 SD)*	
Age (years)	46.3 ± 8.8
Body mass (kg)	84.7 ± 16.1
Body height (m)	1.72 ± 0.11
Body mass index (kg/m ^2^)	28.5 ± 5.2
*Radiographic severity (N (%))*	
KL 1	5 (20.8%)
KL 2	11 (45.8%)
KL 3	7 (29.2%)
KL 4	1 (4.2%)
*Affected side (N (%))*	
Left	10 (41.7%)
Right	14 (58.3%)
*Severity of symptoms (median (interquartile rage))*	
KOOS symptoms	52.5 (38.4–71.4)
KOOS pain	41.7 (30.6–61.8)
KOOS activities of daily living	56.1 (42.6–72.3)
KOOS recreation/sport	22.5 (8.6–37.5)
KOOS quality of life	25.0 (12.5–37.5)
*Quality of life (median (interquartile rage))*	
SF36 total	52.5 (41.3–61.2)
SF36 physical function	50.0 (35.0–65.0)
SF36 pain	33.8 (22.5–47.5)

SD—standard deviation; KL—Kellgren Lawrence grade; KOOS—Knee Osteoarthritis Outcome Score; SF36—Short Form (36) Health Survey.

### Walking stress test

All participants completed a walking stress test at the Functional Biomechanics Laboratory at the Department of Orthopaedics and Traumatology the University Hospital Basel, Switzerland (March 2016 to November 2019). ^33^ Briefly, patients were asked to refrain from physical activity during the 24 hours prior to the experiment and perform minimal walking activity before the study visit. Participants rested in a seated position for 30 minutes before the walking stress. The walking stress comprised walking at self-selected walking speed on a flat treadmill (mercury ^®^ 3p, h/p/cosmos sports & medical GmbH, Nussdorf-Traunstein, Germany). Blood samples were collected immediately before (T0), immediately after (T1), and 0.5h, 1.5h, 3.5h, and 5.5h (T2–T5) after the walking stress. During the walking stress, spatiotemporal parameters (walking speed, cadence, step length) were collected using the built-in pressure plate (Zebris FDM-T, Zebris Medical GmbH, Isny, Germany) and the manufacturer’s software. For the 5.5 hours after the walking stress, participants rested in a seated position to preclude blood volume distribution.

Blood samples (7.5 ml each) were obtained from an antecubital vein. A thin catheter was placed and remained in their vein for 6 hours. As described in the protocol, ^33^ to prevent clogging by clotted blood, we flushed the catheter with 10 ml isotonic saline solution (0.9% NaCl) after every blood draw. The first 3 ml of each sample was discarded to avoid dilution by the injected saline solution. Blood samples clotted in the blood tubes (S-Monovette ^®^ 7.5ml Z-Gel, Sarstedt AG, Nürnbrecht, Germany) at room temperature for 30 minutes. They were then centrifuged (Sarstedt AG &Co SMC6) for 15 minutes at 2016 g, separated into aliquots, frozen (-20°C), and transferred to a -80°C freezer within 48 hours until assays were performed.

After the final blood draw, participants completed gait analysis with a 3-dimensional motion capture system (Vicon Motion Systems Ltd., Oxford, UK) and force plates (Kistler Instrumente AG, Winterthur, Switzerland). We attached reflective skin markers to selected anatomical landmarks according to the Plug-In Gait marker set. ^37^ After collecting data for a standing trial (anatomic upright position), data for three gait trials at self-selected walking speed were collected for each participant (own regular walking shoes; none of the participants used prescribed orthoses or insoles).

### Assessing serum biomarker concentrations

Serum biomarker concentrations were measured using commercial enzyme-linked immunosorbent assays (ELISAs, Table 2). Investigators were blinded to the samples, which were analyzed in duplicate and in random order. Differences due to inter-assay variation were eliminated by comparing concentrations within participants and testing all samples of any participant on the same plate.

**Table 2.** Specifications of the commercial kits used in this study.

Blood marker	Kit name	Vendor	Coefficient of variation (within assay) (%)
COMP	Human COMP protein ELISA kit	BioVendor, Modrice, Czech Republic	2.0
MMP-1	Human Total MMP-1 Immunoassay	R&D Systems Inc., Minneapolis, USA	3.2
MMP-3	Human Total MMP-3 Immunoassay	R&D Systems Inc., Minneapolis, USA	3.1
MMP-9	Human Total MMP-9 Immunoassay	R&D Systems Inc., Minneapolis, USA	2.6
C2C	Human C2C ELISA kit	IBEX Pharmaceuticals Inc., Montréal, Canada	7.4
CPII	Human CPII ELISA kit	IBEX Pharmaceuticals Inc., Montréal, Canada	1.1
IL-6	Human IL-6 Immunoassay	R&D Systems Inc., Minneapolis, USA	1.3
PRG-4	Human Proteoglycan 4 (PRG-4) ELISA Kit	CUSABIO Technology LLC, Houston, USA	5.7
ADAMTS-4	Human ADAMTS-4 ELISA kit	Sigma-Aldrich, Saint Louis, USA	2.4
Resistin	Human Resistin ELISA kit	Mediagnost, Reutlingen, Germany	1.2

COMP—cartilage oligomeric matrix protein; MMP—matrix metalloproteinasis; C2C—fragment resulting from cleavage of type II collagen by collagenases reflecting degradation; CPII—C-propeptide of type II procollagen; IL—interleukin; PRG—proteoglycan; ADAMTS—A disintegrin and metalloproteinase with thrombospondin motifs; ELISA—enzyme-linked immunosorbent assay.

### Assessing accumulated load

Accumulated load was estimated using two different approaches:

(1) Approach 1: Maximum, rate of increase and impulse of the vertical ground reaction force during treadmill walking multiplied by number of steps taken during the walking stress. The vertical ground reaction force was measured for each step using the pressure plate built into the treadmill (Zebris FDM-THM-S pressure plate, Zebris Medical GmbH, Isny, Germany). Vertical ground reaction force trajectories normalized to body weight were computed for each step by the manufacturer’s software and exported. The vertical ground reaction force impulse for each step was calculated as the area under the vertical ground reaction force trajectory during loaded stance phase and normalized to body weight.
(2) Approach 2: Maximum and impulse of the knee adduction moment, knee flexion moment, total knee compressive force, medial and lateral compartment compressive force as well as medio-lateral shear force were calculated from musculoskeletal modeling for the affected knee during overground walking multiplied by number of steps taken during the walking stress. As previously described for the same cohort, ^38^ kinematic and ground reaction force (GRF) data were filtered using a second-order low-pass Butterworth filter with a cut-off frequency of 5 Hz and 12 Hz, respectively. Gait events were determined from the GRF measurements using force thresholds (>20 N for foot-strike and <20 N for foot-off). Marker trajectories and GRF data were used as input for an inverse dynamics analysis in the AnyBody Modelling System (AnyBody Technology A/S, Aalborg, Denmark). Personalized models for each subject were created as previously described ^39^ from a detailed generic model of the lower limb ^40^ (based on a cadaveric dataset ^41^) scaled to match the overall anthropometrics to each subject and the marker data collected during the standing reference trial of the same subject. ^42^ We computed joint kinematics from the measured marker trajectories, and calculated the required muscle activations, resulting knee total, flexion and adduction moments, as well as the proximo-distal compressive force, medial and lateral compartment compressive force, and medio-lateral shear force by an inverse dynamics analysis based on a third-order-polynomial muscle recruitment criterion. ^43^ All moments were reported as external net moments, and all forces and moments were normalized to body weight. The impulse of each variable was calculated as the area under the curve during loaded stance phase for each step.

### Statistical analysis

For the biomarkers PRG-4 and ADAMTS-4, 23 and 63 measurements were below the detection limit, respectively. We imputed these measurements by using the midpoint between 0 and the lower detection limit. ^44^

Load-induced changes in biomarkers were defined as

$$∆{\text{biomarker}}_{b-a}=\text{biomarker}\left(\mathrm{Tb}\right)-\text{biomarker}\left(\mathrm{Ta}\right)$$

where $a,\mathrm{b\epsilon}\left[0,1,2,3,4,5\right]$ and b>a. Normality assumptions were violated for all biomarkers, except COMP, according to the Shapiro-Wilk test. We used a non-parametric test (Wilcoxon signed rank test) that does not rely on assumptions of absence of outliers and normality of the data to compare pairwise biomarker concentrations. Nonparametric descriptive statistics including median and interquartile range (IQR) were computed for each marker and each time point. Spearman correlation coefficients were used to detect correlations among load induced changes in blood levels among blood markers across time points and with patient reported osteoarthritis outcome and QoL, load during each step, and load accumulated during the walking stress.

To assess the association between accumulated load and load-induced blood marker kinetics, relative changes in blood markers were computed as

$${\text{biomarker}}_{\text{TbrT}0}=\frac{\text{biomarker}\left(\mathrm{Tb}\right)}{\text{biomarker}\left(T0\right)}*100$$

where $\mathrm{b\epsilon}\left[1,2,3,4,5\right]$ . Spearman correlation coefficients were used to detect correlations of blood markers concentrations at T0 with load during each step and to detect correlations of relative blood markers kinetics with load accumulated during the walking stress.

We only interpreted associations between blood marker levels and their load-induced changes with patient reported osteoarthritis outcome and QoL or loading parameters if significant correlations were observed with at least two KOOS or SF36 subscores or at least two loading parameters to account for the possibility of detecting correlations by chance.

ADAMTS-4 was log transformed to address skewness. Because we intended to only interpret results that are in concordance with previous evidence for mechanosensitivity, ^45^ we refrained from applying Bonferroni corrections and set the significance level to 0.05. Statistical analysis was performed in R version 4.0.3 (The R Foundation, Vienna, Austria). Assuming that a true correlation has an absolute value of 0.60 or greater (0.60 being considered an overall moderate correlation), a sample size of 19 is required to determine if the correlation coefficient differs from zero with a power of 80% and an alpha significance level of 5%.

## Results

During the 30-minute walking stress, patients walked at a median speed of 0.94 (IQR, 0.70–1.04) m/s with a cadence of 100.6 (92.1–107.3) steps/minute and step length of 0.56 (0.45–0.61) m, and took 2416 (1410–2945) steps. Descriptive statistics of the loading parameters per step and the estimated accumulated load are shown in Table 3. Detailed loading trajectories for a subset of patients have been published previously. ^39^

**Table 3.** Median (interquartile range) load per step and estimated accumulated load during walking stress test.

Loading parameter	Load per step	Accumulated load Load per step * number of steps
*Treadmill walking during stress test*		
Vertical ground reaction force		
Peak (%BW)	96.5 (88.8–102.7)	282881 (245312–314139)
Impulse (BW*s)	0.55 (0.50–0.58)	1628 (1552–1679)
*Detailed ambulatory biomechanics analysis (overground)*		
Knee flexion moment		
Peak (Nm/BW)	0.36 (0.19–0.54)	1060 (562–1665)
Impulse (Nm/BW*s)	0.07 (0.02–0.12)	204 (57–351)
Knee adduction moment		
Peak (Nm/BW)	0.50 (0.40–0.59)	1426 (1119–1899)
Impulse (Nm/BW*s)	0.20 (0.14–0.25)	584 (388–811)
Total knee moment		
Peak (Nm/BW)	0.63 (0.51–0.80)	1769 (1529–2422)
Impulse (Nm/BW*s)	0.31 (0.27–0.39)	934 (750–1104)
Medial compartment compressive force		
Peak (BW)	2.28 (2.03–2.62)	6703 (5818–8323)
Impulse (BW*s)	1.22 (1.11–1.43)	3638 (3259–4266)
Lateral compartment compressive force		
Peak (BW)	0.78 (0.58–1.00)	2377 (1739–3162)
Impulse (BW*s)	0.46 (0.33–0.59)	1361 (971–1707)
Knee medio-lateral shear force		
Peak (BW)	0.69 (0.56–0.80)	2111 (1427–2557)
Impulse (BW*s)	0.27 (0.22–0.33)	780 (655–1009)
Total joint compressive force		
Peak (BW)	2.83 (2.60–3.35)	8711 (7373–10128)
Impulse (BW*s)	1.76 (1.60–1.93)	5178 (4644–5804)
Vertical ground reaction force		
Peak (%BW)	110.0 (105.8–114.0)	328071 (281442–363652)
Impulse (BW*s)	0.56 (0.54–0.61)	1654 (1530–1757)

BW—body weight.

### Blood marker kinetics in response to load

**Figure 1.**
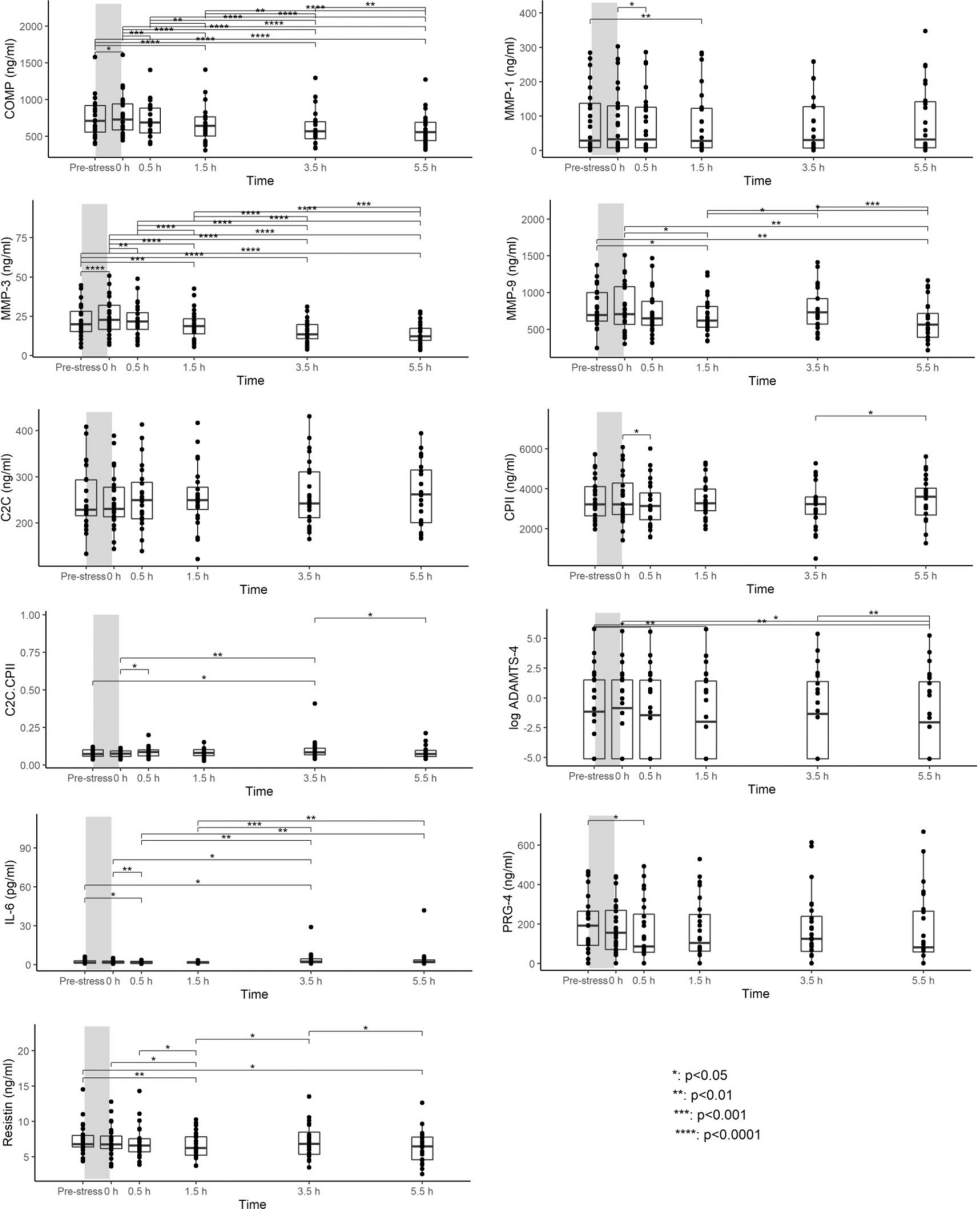
Serum concentrations at each of six time points immediately before (T0) and 0h (T1), 0.5h (T2), 1.5h (T3), 3.5h (T4) and 5.5h (T5) after the walking stress. Boxplots show median, 25% and 75% quartiles and individual data points. *p<0.05; **p<0.01; ***p<0.001; ****p<0.0001. Grey bars illustrate the walking stress.

A summary of each blood marker at the different time points is given in the extended data 1. ^65^ Compared to pre-walking stress serum concentrations, COMP and MMP-3 levels differed significantly between time points. Levels increased from pre-stress to 0h, returned to pre-stress levels within 0.5h and then continued to decrease further until 5.5h post walking stress ( Figure 1). MMP-9 and resistin levels were significantly lower at 1.5h and 5.5h post walking stress than pre-stress levels ( Figure 1). Although significant differences in ADAMTS-4 and IL-6 between several time points were observed, there was no clear pattern of a loading response ( Figure 1). Few to no significant differences in MMP-1, C2C, CPII, C2C/CPII, and PRG-4 levels between time points were observed. As a sensitivity analysis, we excluded measurement for ADAMTS-4 and PRG-4 that were below the detection limit. Results were coherent with the results from the imputed dataset. Please note that a subset of this data has been previously presented at a conference. ^46^

### Correlations among load-induced changes in blood levels across time points

**Figure 2.**
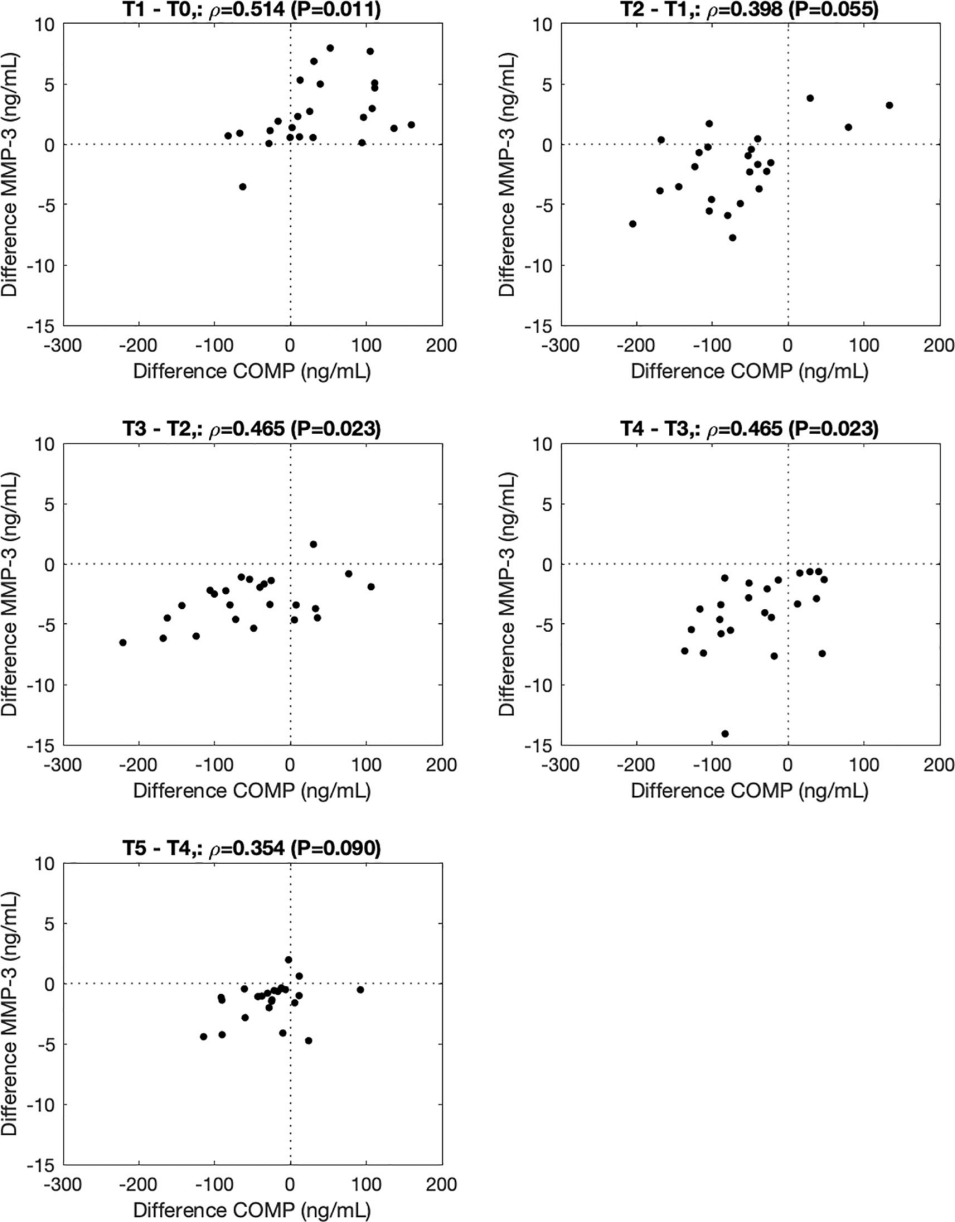
Association between changes in COMP and MMP-3 from before to immediately after (-0.5h to 0h; T1-T0), and 0h to 0.5h (T2-T1), 0.5h to 1.5h (T3-T2), 1.5h to 3.5h (T4-T3), and 3.5h to 5.5h (T5-T4) after the walking stress.

**Figure 3.**
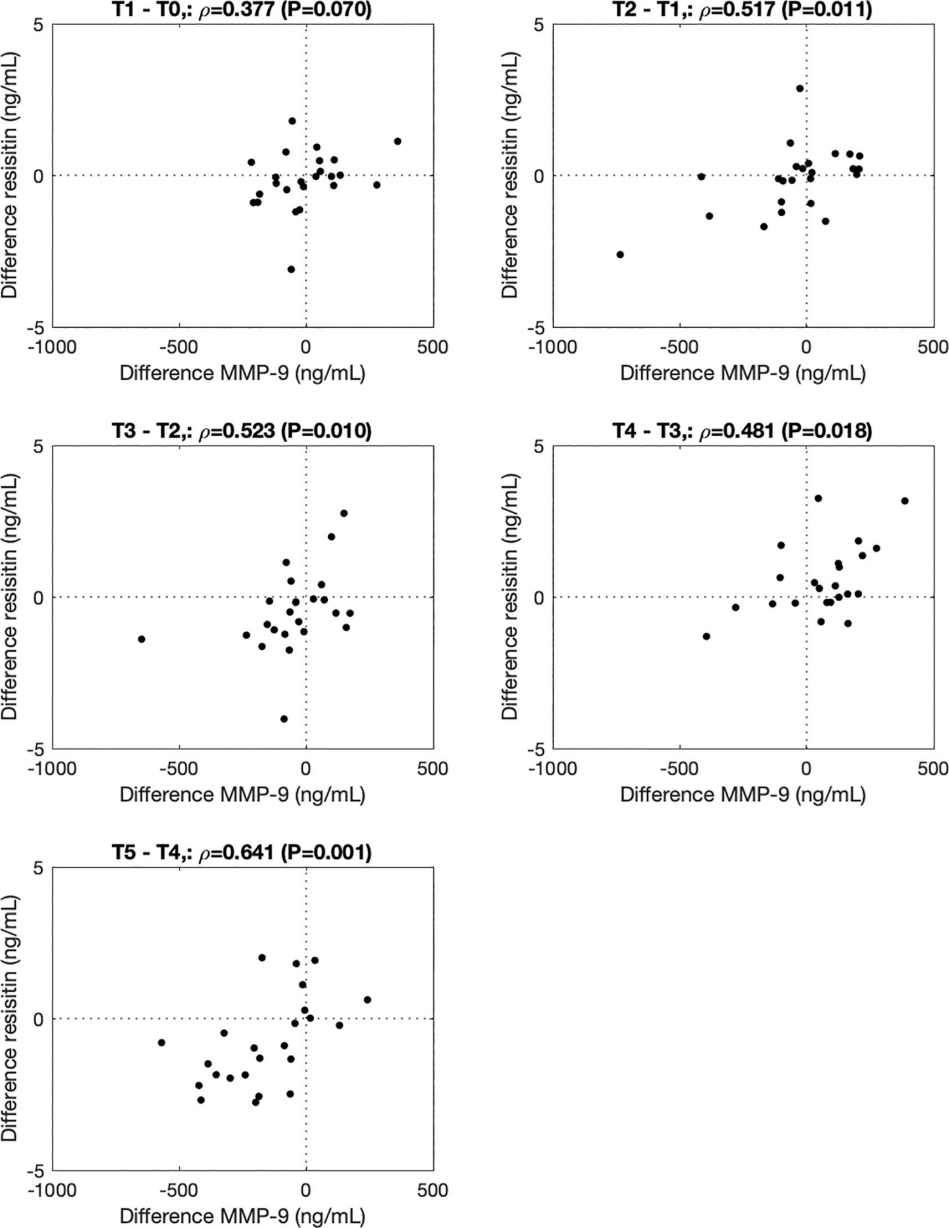
Association between changes in MMP-9 and resistin from before to immediately after (-0.5h to 0h; T1-T0), and 0h to 0.5h (T2-T1), 0.5h to 1.5h (T3-T2), 1.5h to 3.5h (T4-T3), and 3.5h to 5.5h (T5-T4) after the walking stress.

∆COMP correlated with ∆MMP-3 from before to immediately after (T1-T0, ρ=0.514, P=0.011), from 0.5h to 1.5h (T3-T2, ρ=0.465, P=0.023), and from 1.5h to 3.5h (T4-T3, ρ=0.465, P=0.023) after the walking stress ( Figure 2). ∆MMP-9 correlated with ∆resistin from 0h to 0.5h (T2-T1, ρ=0.517, P=0.011), 0.5 to 1.5h (T3-T2, ρ=0.523, P=0.010), 1.5h to 3.5h (T4-T3, ρ=0.480, P=0.018) and 3.5h to 5.5h (T5-T4, ρ=0.641, P=0.001) after the walking stress ( Figure 3).

**Figure 4.**
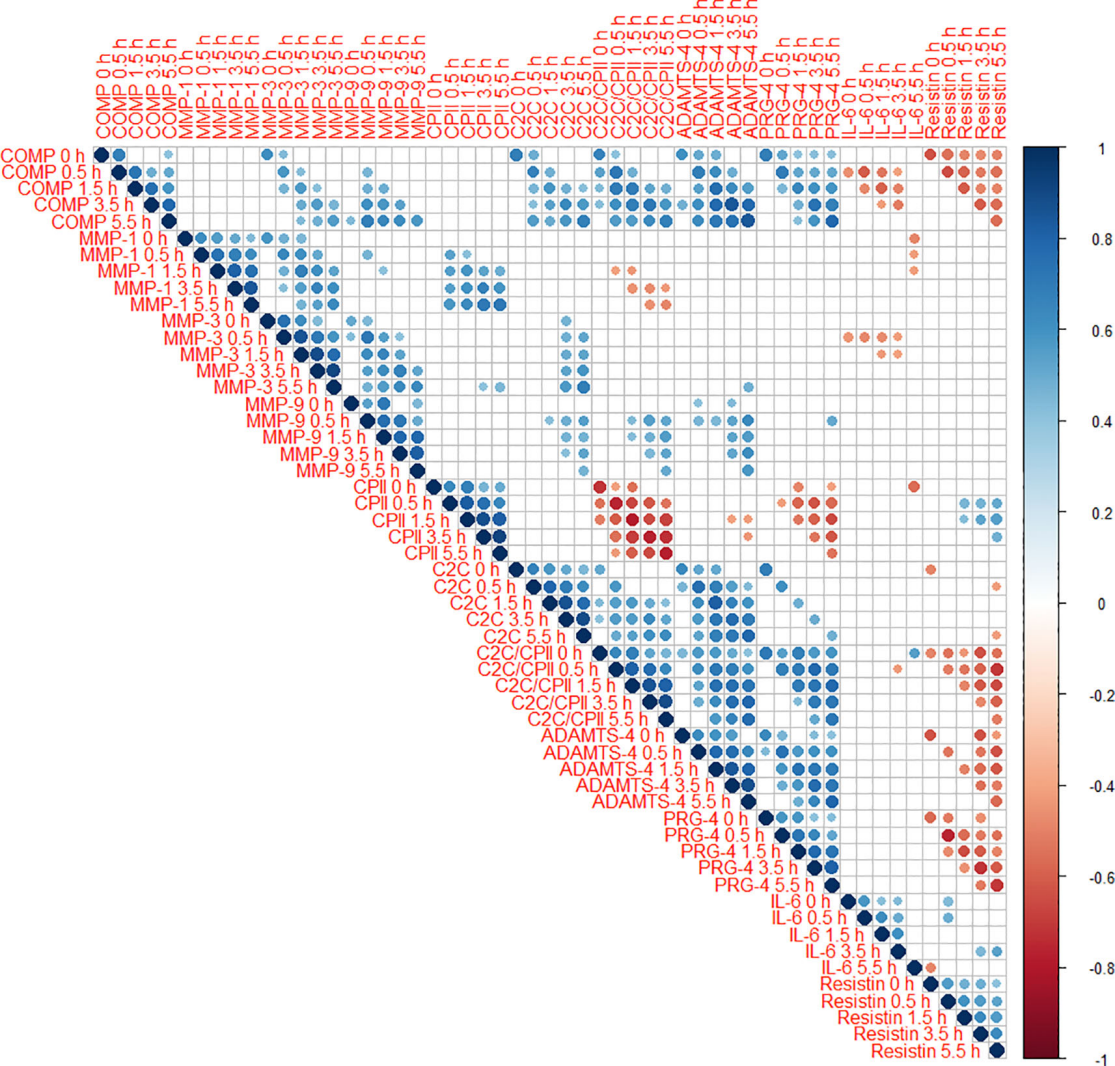
Correlogram among change from baseline (T0) to 0h (T1), 0.5h (T2), 1.5h (T3), 3.5h (T4) and 5.5h (T5) of the different blood markers. Positive correlations are displayed in blue and negative correlations in red color. Color intensity and the size of the circle are proportional to the correlation coefficients. The legend color shows the correlation coefficients and the corresponding colors. Only statistically significant correlations are shown (P<0.05).

Cross-correlations of change from baseline concentrations (T0) among all markers and time points (T1 to T5) are shown as correlogram in Figure 4. ∆COMP from baseline correlated positively with ∆MMP-3, ∆C2C, ∆CPII, ∆ADAMTS-4, ∆PRG-4 and ∆C2C/CPII ratios from baseline and negatively with ∆IL-6 and ∆resistin from baseline for most time point comparisons. Several other positive and negative correlations among change from baseline among markers and time points were observed ( Figure 4).

### Correlation of baseline marker levels and load during each step

**Figure 5.**
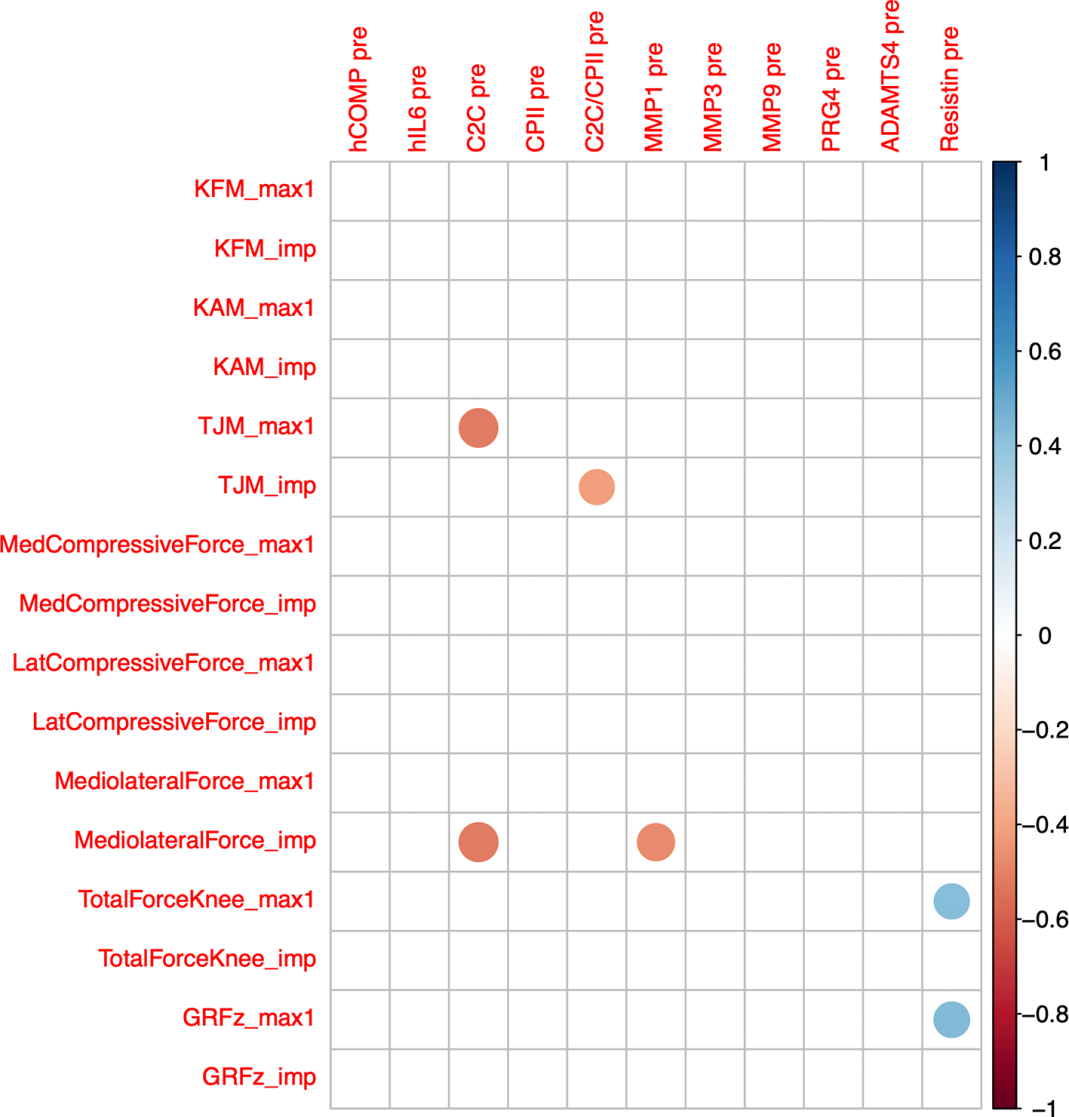
Correlogram between baseline (T0) blood marker concentrations and loading parameters for a single gait cycle. Max1—first maximum of loading parameter trajectory during gait cycle; imp—impulse of loading parameter for one gait cycle (=integral of loading parameter over time); GRF—ground reaction force; PBW—per body weight; KFM—knee flexion moment; KAM—knee adduction moment; TJM—total joint moment; med—medial; lat—lateral. Color intensity and the size of the circle are proportional to the correlation coefficients. The legend color shows the correlation coefficients and the corresponding colors. Only statistically significant correlations are shown (P<0.05).

C2C at T0 correlated negatively with the peak total joint moment (ρ=-0.51, P=0.013) and the knee medio-lateral shear force impulse (ρ=-0.52, P=0.011; Figure 5). C2C/CPII correlated negatively with the total joint moment impulse (ρ=-0.42, P=0.042). MMP1 at T0 correlated negatively with the knee medio-lateral shear force impulse (ρ=-0.47, P=0.023). Resistin at T0 correlated positively with the first peak knee total compressive force (ρ=0.42, P=0.045) and vertical ground reaction force (ρ=0.43, P=0.033). None of the other blood marker concentrations at T0 correlated with any parameter describing load exerted during a single step.

### Correlation of load-induced blood marker kinetics and load accumulated during the walking stress

**Figure 6.**
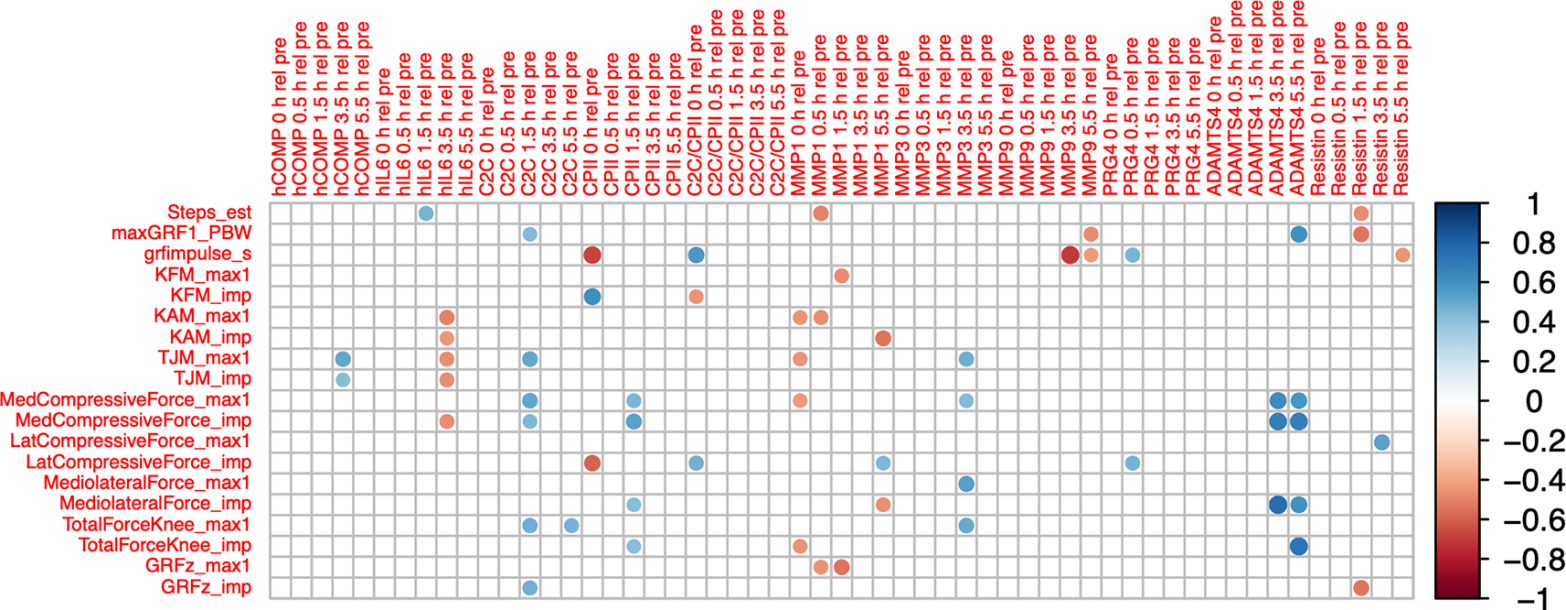
Correlogram between relative change of blood marker concentration from baseline (T0) for all time points (T1 to T5) and accumulated load during 30-minute walking stress (loading parameters for a single gait cycle multiplied by number of steps). Max1—first maximum of loading parameter trajectory during gait cycle; imp—impulse of loading parameter for one gait cycle (=integral of loading parameter over time); GRF—ground reaction force; PBW—per body weight; KFM—knee flexion moment; KAM—knee adduction moment; TJM—total joint moment; med—medial; lat—lateral. Color intensity and the size of the circle are proportional to the correlation coefficients. The legend color shows the correlation coefficients and the corresponding colors. Only statistically significant correlations are shown (P<0.05).

COMP _T4rT0_ correlated positively with accumulated peak total joint moment (ρ=0.52, P=0.014) and accumulated total joint moment impulse (ρ=0.43, P=0.047; Figure 6). IL6 _T4rT0_ correlated negatively with accumulated peak knee adduction moment (ρ=-0.49, P=0.020) and impulse (ρ=-0.43, P=0.045), with accumulated peak total joint moment (ρ=-0.47, P=0.028) and impulse (ρ=-0.46, P=0.031), and with accumulated medial compartment compressive force impulse (ρ=-0.46, P=0.030). C2C _T34rT0_ correlated positively with accumulated peak total joint moment (ρ=0.52, P=0.014), peak medial compartment compressive force (ρ=0.52, P=0.012) and impulse (ρ=0.45, P=0.038), peak total joint force (ρ=0.48, P=0.023), and ground reaction force impulse (ρ=0.48; P=0.024). CPII _T1rT0_ correlated negatively with accumulated vertical ground reaction force impulse (ρ=-0.67, P=<0.001) and lateral compartment compressive force impulse (ρ=-0.58, P=0.005), and positively with accumulated knee flexion moment impulse (ρ=0.62, P=0.002). CPII _T3rT0_ correlated positively with accumulated peak medial joint force (ρ=0.45, P=0.035) and impulse (ρ=0.53, P=0.011), medio-lateral shear force impulse (ρ=0.43, P=0.047) and total compressive force impulse (ρ=0.43, P=0.047). MMP1 _T1rT0_ correlated negatively with accumulated peak knee adduction moment (ρ=-0.44, P=0.039), peak total joint moment (ρ=-0.44, P=0.039), peak medial compartment compressive force (ρ=-0.43, P=0.043) and total compressive force impulse (ρ=-0.44, P=0.039). MMP1 _T5rT0_ correlated negatively with accumulated knee adduction moment impulse (ρ=-0.54, P=0.010) and knee medio-lateral shear force impulse (ρ=-0.45, P=0.036), and positively with accumulated lateral compartment compressive force impulse (ρ=0.45, P=0.036). MMP3 _T4rT0_ correlated positively with accumulated peak total joint moment (ρ=0.49, P=0.021), peak medial compartment compressive (ρ=0.44, P=0.043), medio-lateral shear (ρ=0.54, P=0.009) and total compressive force (ρ=0.51, P=0.015). Correlations of ADAMTS5 were not further considered because of the high number of imputed data points.

### Correlations of load-induced blood marker kinetics with patient reported osteoarthritis outcome and QoL

C2C levels before the walking stress (T0) correlated negatively with the KOOS subscales symptoms (ρ=-0.439, P=0.032), pain (ρ=-0.503, P=0.012), activities of daily living (ρ=-0.607, P=0.002), and recreation/sports (ρ=-0.677, P<0.001; extended data 2 (65)). Patients with higher C2C levels had lower osteoarthritis outcome scores (greater pain respective limitations). ∆IL-6 (T1-T0) correlated positively with the total SF36 score (ρ=0.430, P=0.036) and the SF36 category physical function (ρ=0.467, P=0.022; extended data 3 (65)). Patients with a smaller load induced change in IL-6 had lower SF36 scores. ∆MMP-3 (T1-T0) correlated positively with the KOOS subscales QoL (ρ=0.429, P=0.036), and with the SF-36 category pain (ρ=0.444, P=0.030). Patients with a smaller load induced change in MMP-3 had lower QoL scores (greater pain respective limitations).

## Discussion

The purpose of this exploratory study was (i) to quantify the mechanoresponse of this array of potential blood markers for joint pathology (COMP, MMP-1, MMP-3, MMP-9, CPII, C2C, C2C/CPII, ADAMTS-4, PRG-4, IL-6 and resistin) to a walking stress test in patients with knee OA and to determine the correlation (ii) among the kinetics of these blood markers, (iii) with accumulated knee load during the walking stress, and (iv) with patient reported osteoarthritis outcome and QoL. Only COMP and MMP-3 showed a consistent increase immediately after the walking stress while COMP, MMP-3, MMP-9 and resistin all showed consistent delayed decreases below pre-stress levels up to 5.5h post walking stress. COMP and MMP-3 kinetics in response to the mechanostimulus correlated as did load-induced MMP-9 and resistin kinetics. Moreover, we observed correlations of change from baseline levels among several blood markers for several time points, between baseline levels and load per gait cycle, between load-induced changes in selected blood markers and accumulated load during the walking stress, and between baseline levels and load-induced changes in selected blood markers with patient reported osteoarthritis outcome and QoL.

The results are in line with previous reports of an initial increase in COMP above pre-stress levels during a 30-minute walking exercise ^2^^–^^4^^,^^6^^–^^15^ followed by – on average – a continuous decrease in COMP well below pre-stress levels in the 5.5 hours after the walking stress but with a large variability in patients with OA and in older adults. ^3^^,^^4^ Previous work including a control experiment where COMP remained stable has shown that the increase in COMP after the walking stress is clearly attributed to the walking stress. ^2^ The pattern of a prolonged decrease contrasts observations of the mechanoresponse of COMP in a walking stress test in young healthy adults. ^2^ The consistent results in patients with knee OA are particularly relevant as the delayed mechanoresponse of COMP has been shown to predict future changes in knee cartilage morphology in patients with OA ^4^ and in older adults. ^47^ Erhart-Hledik *et al.* ^4^ reported that in 17 patients with medial compartment knee OA changes in COMP levels from pre-stress levels to 3.5h and 5.5h after the walking stress varied largely among patients (ranging from 40% decrease to 40% increase) and that those with higher COMP levels 3.5h and 5.5h after the walking stress experienced a greater loss in articular cartilage five years later. Moreover, in that study neither pre-stress COMP levels nor changes in COMP levels immediately after the walking stress correlated with cartilage thickness changes over five years.

In another study on 12 asymptomatic persons aged 45 years and older, changes in COMP levels from pre-stress levels to 5.5h after the walking stress varied largely among persons (ranging from 54% decrease to 22% increase) and that those with higher COMP levels 5.5h after the walking stress experienced a greater loss in articular cartilage seven to nine years later. ^47^ This association was not as strong as that in patients with medial compartment knee OA. ^4^ These results suggest that patients with knee OA and older asymptomatic adults show a large variability in load induced COMP kinetics and that those with prolonged elevated COMP levels will experience greater cartilage degeneration in subsequent years. However, the factors determining the kinetic patters are not understood and it is unknown how load induced COMP kinetics evolve throughout a person’s lifetime. In our study, baseline COMP did not correlate with joint load during one gait cycle but patients with greater accumulated load during the walking stress still had higher relative COMP levels 3.5 hours after the walking stress suggesting the relevance of the characteristics of a mechanostimulus for of the response of COMP to this stimulus. This result is in agreement with a recent study showing that the response of blood markers (i.e. type II collagen degradation and formation-degradation ratio) to exercise depends on characteristics of the exercise (impact—running versus no impact—cycling). ^48^ Overall, COMP kinetics in response to a walking stress appear may be determined not only by the presence of OA or the specific mechanostimulus but also influenced by age as previously suggested, ^3^ and relevant for degenerative processes of articular cartilage.

Similar kinetics of COMP and MMP-3 and correlations with accumulated load observed here are in line with previous reports in studies on the response of blood biomarkers to extreme ambulatory exercise ^49^ and to immobilization during bed rest. ^50^^,^^51^ Changes in COMP were linearly related with changes in MMP-3 throughout an ultramarathon race (4486-km during 64 running days without any rest days) where in 68% of runners, ultramarathon-induced changes in MMP-3 levels explained more than 30% of ultramarathon-induced changes in COMP levels. ^49^ Liphardt *et al.* showed in two separate studies involving 5-day ^51^ respective 21-day ^50^ bedrest under highly controlled conditions that COMP and MMP-3 decreased by 10 to more than 20% within the first 24 hours of bedrest and both returned to pre-bedrest levels within 24 hours after bedrest. Moreover, COMP and MMP-3 levels in serum respective in synovial fluid were higher in patients with diagnosed knee OA and that patients with more severe knee OA had higher COMP and MMP-3 levels than patients with less severe knee OA. ^52^^,^^53^ MMP-3 activates other MMPs such as MMP-1 and MMP-13, ^54^ which may lead to an initial high release of type II collagen fragments ^55^ and COMP cleavage. ^56^ The strong correlation among the load-induced kinetics of COMP and MMP-3 and with the accumulated load in our study and their metabolic relationship suggest that COMP and MMP-3 may not only be involved in the pathophysiology of OA but also that MMP-3 may play a role in regulating COMP according to mechanical demands. The latter is further supported by our finding that the load induced changes in MMP-3 correlated with patient reported osteoarthritis outcome and QoL.

Interestingly, MMP-9 showed similar load-induced kinetics as resistin, and both had a tendency towards a negative correlation with accumulated load. This pattern clearly differed from that of COMP and MMP-3 without an immediate but with a delayed response. We did not observe an association of MMP-9 and resistin levels at baseline. Hence, the similar patterns in load-induced kinetics can be clearly attributed to the walking stress. In contrast, a more than twofold increase in resistin (and association with load-induced changes in MMP-3) in lean adults has been reported for marathon running. ^57^ Clearly, marathon running represents a much greater physiological stress than walking for 30 minutes. Resistin levels are higher in obese persons and in patients with severe knee OA. ^58^ Moreover, resistin may lead to an overexpression of MMPs. ^28^^,^^29^ Hence, a decrease in resistin (and MMP-9) levels elicited by daily activities such as walking may be particularly relevant in patients with OA and/or obesity. In our study, we observed a decrease in resistin and MMP-9 levels in response to walking stress. This finding may reflect stress-induced suppression of resistin and downregulation of MMP-9, which could indicate a chondroprotective function of exercise by impeding the proteolytical digestion of matrix components such as gelatin. The potential relevance of this mechanoresponse is further supported by the observation that associations of changes in response to load for COMP, ADAMTS-4, and PRG-4 levels and C2C/CPII ratios with resistin and their correlations with accumulated load only emerged 0.5h after the walking stress.

In our study, the walking stress did not elicit consistent changes in kinetics for MMP-1, C2C, CPII, C2C/CPII, IL-6, PRG-4, and ADAMTS-4. Previous evidence suggested that COMP, MMP-3, MMP-9, ADAMTS-4, CPII and IL-6 could suitable for assessing in vivo cartilage mechanosensitivity. ^45^ It is possible that these markers may reflect individual disease progression despite of the lack of group results. Joint load assessed during gait analysis can be considered a surrogate of the typical loading of an individual’s joint. The observed negative correlations of catabolic blood markers (MMP1, C2C) with joint load during gait assessed using gait analysis may suggest that those patients who have lower joint level loads tend to have higher joint metabolism or – as indicated by C2C/CPII – a greater misbalance towards cartilage destruction. These results agree with previous evidence on the role of underloading in early knee OA after an ACL injury. ^59^

Interestingly, we did not observe a clear pattern of loading response of IL-6 to the walking stress nor an association with any of the other blood makers except a moderate correlation with the response to load for COMP and MMP-3 at time points after the walking stress. Almost all patients included in this experiment had mild to moderate OA with moderate symptoms. The data on IL-6 are not sufficient to rule out the possibility that inflammation played a role in the current disease process of this population or in their metabolic response to the walking stress. For instance, Atkinson *et al.* ^60^ have recently shown the presence of local inflammation using knee effusion-synovitis volume and a correlation between the change in the load-distribution and change in knee effusion-synovitis volume in patients undergoing high tibial osteotomy suggesting the phenomenon of mechano-inflammation in patients with knee OA. While such data cannot be retrospectively produced in the current study, future analyses may assess other inflammatory markers to further elucidate the role of inflammation in this population.

We observed an association of load-induced kinetics among these blood markers and the association of C2C and ∆MMP-3 (both markers of tissue degradation) with patient reported osteoarthritis outcome and QoL. These associations may provide novel insights into disease activity and metabolic processes. The association of C2C with patient reported osteoarthritis outcome and QoL is consistent with previous reports of negative correlations of urine C2C levels with KOOS subscores in women with knee OA. ^61^ Moreover, urinary C2C was one of the markers predictive of worsening pain and radiographic OA over 2 years. ^62^ C2C levels also differ between patient with multijoint and those with single-joint OA ^63^ and correlate with disease severity as determined by magnetic resonance imaging. ^64^ The average C2C levels in patients with knee OA in our study were comparable to those obtained in healthy athletes. ^65^ The negative correlation between C2C levels and patient-reported OA outcomes suggests that patients with lower OA outcome scores have higher catabolic activity, possibly reflecting increased tissue turnover or tissue degradation. Further insight into this association could be obtained in patients undergoing treatment for knee OA, including joint-preserving procedures such as corrective osteotomy.

MMP-3 consistently changes with ambulatory load and immobilization ^45^^,^^49^^–^^51^ and is relevant for OA. ^52^^,^^53^ Our finding of an association between ∆MMP-3 and patient reported outcome measures supports the importance of MMP-3 in the context of cartilage mechanosensitivity. MMP-3 degrades collagen types II, III, IV, IX, and X, proteoglycans, fibronectin, laminin, and elastin ^21^ and is thus critical for tissue turnover. MMP-3 is also involved in metabolic processes in other tissues than articular cartilage. Nevertheless, higher load-induced systemic concentrations may influence healthy or pathological cartilage metabolism. The dose-response of load-induced changes in MMP-3 was stronger than those in COMP in healthy persons. ^45^ Most studies on in vivo mechanosensitivity of articular cartilage in health and disease have focused on COMP. ^2^^–^^4^^,^^6^^–^^15^ Greater ambulatory load lead to a greater load-induced increase in MMP-3 in healthy persons. ^45^ Our result that patients with a higher QoL had a greater load-induced increase in MMP-3 suggests that metabolic processes are related to QoL. Our observations of correlations of blood marker kinetics with the accumulated load during the walking stress emphasizes the need to quantify and consider these parameters in future studies employing loading stress tests. In particular, immediate or delayed positive correlations of some blood markers (C2C, CP2, MMP3, PRG4) with accumulated load and negative correlations of others (IL6, MMP1, MMP9) point towards differential metabolic response to a walking stress. Future longitudinal studies in patients with knee OA are warranted to shed light on the relevance of this finding in the context of future disease progression.

### Limitations

Participants were instructed to minimize their physical activity during the 24 hours prior to the experiment but their activity more than 30 minutes prior to the start of the walking stress was not monitored, and hence a potential effect of pre-stress physical activity cannot be excluded. Participants walked at their preferred speed for 30 minutes, and the speed and hence the number of steps taken during the walking stress differed among participants. We decided to prescribe the length of the physical stress rather than the number of loading cycles as to date the relevance of one over the other on the metabolic response to a mechanical stimulus is unknown and to facilitate comparison with the literature. However, we addressed this presumed limitation by estimating the load accumulated during the walking stress test and assessing the effect of the accumulated load on blood marker kinetics in response to load. Blood markers reflect contributions of each marker from all joints in the body, and it is thus possible that the serum biomarker kinetics presented here reflect the effects of walking stress on all joints, not just the knee affected by OA. Previous studies ^4^^,^^47^ showing a correlation of load-induced blood marker kinetics (e.g., COMP) with subsequent changes in articular cartilage thickness. Moreover, even if elevated blood markers (e.g., enzymes) in response to load may originate from other tissues, their systemic presence may still affect articular cartilage. These considerations suggest that the results reported here are relevant despite these limitations. Here, we report on selected blood markers for joint pathology using ELISAs. Applying other approaches such as untargeted or targeted metabolomics or proteomics to a controlled experiment as presented here may be useful for identifying other blood markers relevant for in vivo mechanobiology of articular cartilage in health and pathology.

## Conclusion

This exploratory study represents a comprehensive analysis of the in vivo response of several blood markers of joint pathology to a mechanical stimulus and novel insights into associations of load-induced blood marker kinetics and accumulated load. Changes in blood marker levels in response to load correlated among COMP, MMP-1, MMP-3, MMP-9, CPII, C2C, CPII/C3C, IL-6, ADAMTS-4, PRG-4, and resistin. Similar load-induced kinetics of COMP and MMP-3 as well as those of MMP-9 and resistin, respectively, suggest the presence of distinct and differentiated metabolic responses to a walking stress. While the data presented here cannot be directly linked to pathophysiological processes, the associations with accumulated load during the walking stress and with patient reported osteoarthritis outcome and QoL emphasize the relevance of not only assessing concentrations of (single) blood biomarkers but also considering the mechanoresponse of (arrays of) blood markers in the context of OA pathomechanics.

### Ethical considerations

The study was approved by the regional ethics board (August 19, 2015; Ethikkommission Nordwest- und Zentralschweiz EKNZ 2015-224) and conducted in accordance with the Declaration of Helsinki, and participants provided written informed consent prior to participation.

## Author contributions

AM designed the study. CN recruited the participants and collected the data; EDP conducted the musculoskeletal modeling computation; CN, SH and FC prepared the data for statistical analysis; FC performed the statistical analysis; AM, SH, AML, CN and CE were involved in data interpretation; AM, SH and CN prepared the manuscript; AM, CN, AML, SH, FC, EDP and CE contributed to reviewing and revising the manuscript, and approved the final draft.

## Data Availability

Zenodo: Patient reported outcome measures, load-induced blood marker kinetics, and ambulatory knee load in patients with medial knee compartment osteoarthritis, https://doi.org/10.5281/zenodo.7648802. ^66^ This project contains the following underlying data:
•
Mastertable_all_pre.xls: Patient demographics, patient reported outcome subscores, raw concentrations for each marker (COMP, MMP-1, MMP-3, MMP-9, CPII, C2C, C2C/CPII, ADAMTS-4, PRG-4, IL-6 and resistin), parameters describing walking stress, and ambulatory knee load; metadata describing all parameters. Zenodo: Patient reported outcome measures, load-induced blood marker kinetics, and ambulatory knee load in patients with medial knee compartment osteoarthritis, https://doi.org/10.5281/zenodo.7648802. ^66^ This project contains the following extended data:
• Extended_data.pdf: Median (interquartile range) serum concentrations for all time points; cross-correlations between baseline blood marker levels and patient reported outcome measures; and cross-correlations between load-induced change in blood marker levels and patient reported outcome measures. Data are available under the terms of the Creative Commons Attribution 4.0 International license (CC-BY 4.0). TREND checklist for ‘Load-induced blood marker kinetics in patients with medial knee compartment osteoarthritis are associated with accumulated load and patient reported outcome measures’, https://doi.org/10.5281/zenodo.7648802. ^66^
