# Supplementary material for: Load-induced blood marker kinetics in patients with medial knee compartment osteoarthritis are associated with accumulated load and patient reported outcome measures
Source: F1000Res. 2024 Jan 12;12:299. Originally published 2023 Mar 17. [Version 2] doi: 10.12688/f1000research.131702.2 (PMC11179051; doi:10.12688/f1000research.131702.2)
Supplement: Peer review history

# Reviewer report: Reviewer response for version 2

**DOI:** 10.5256/f1000research.161249.r289957

**Published:** 2024-06-14

**Authors:** Jiacan Su

**Affiliations:** 1 Shanghai University, Shanghai, China

This article has a comprehensive discussion in the field of Load-induced blood marker kinetics in patients with medial knee compartment osteoarthritis. The experimental design is concise and the statistical method is accurate. This article provides new clinical evidence for the filed of mechanical force and osteoarthritis. However, some revisions would prove this article.

 1. It would be better to draw the fit curve in the figure 2 and figure 3.

 2. In the section of “Correlations of load-induced blood marker kinetics with patient reported osteoarthritis outcome and QoL”, the author did not clarify the relationship between other markers with osteoarthritis estimated systems. It is unclear whether the non-mentioned markers have an insignificant relationship with osteoarthritis estimated systems, like SF36.

 3. In the section of “Correlations among load-induced changes in blood levels across time points”, it would be better to explain why only ΔCOMP/ΔMMP-3 and ΔMMP-9/Δresistin was presented in the article.

 4. This article has a deep discussion of medial knee compartment osteoarthritis. All the conclusions and results need to be confined to “medial knee compartment osteoarthritis”. It not appropriate to expand the conclusions and results to “osteoarthritis”.

 5. Here are some articles that are related to author`s article. Hoping they could provide some references for the author.[1],[2]

 https://www.science.org/doi/10.1126/sciadv.abo7868

 https://www.researchgate.net/publication/362046540_Biomaterials_Translational_-_The_New_Vehicle_for_Translational_Medicine

 https://www.sciencedirect.com/journal/cell-reports-medicine/vol/4/issue/10

Is the work clearly and accurately presented and does it cite the current literature?

Yes

If applicable, is the statistical analysis and its interpretation appropriate?

Yes

Are all the source data underlying the results available to ensure full reproducibility?

Yes

Is the study design appropriate and is the work technically sound?

Yes

Are the conclusions drawn adequately supported by the results?

Yes

Are sufficient details of methods and analysis provided to allow replication by others?

Yes

Reviewer Expertise:

Osteoarthritis、Osteoporosis、Fracture、Biomaterials

I confirm that I have read this submission and believe that I have an appropriate level of expertise to confirm that it is of an acceptable scientific standard, however I have significant reservations, as outlined above.

## References

[1] : Osteoarthritis animal models for biomaterial-assisted osteochondral regeneration. *Biomater Transl* .2022;3(4) : 10.12336/biomatertransl.2022.04.006 264-279 10.12336/biomatertransl.2022.04.006 36846505 PMC9947734

[2] : MMP13-targeted siRNA-loaded micelles for diagnosis and treatment of posttraumatic osteoarthritis. *Bioact Mater* .2024;37: 10.1016/j.bioactmat.2024.04.010 378-392 10.1016/j.bioactmat.2024.04.010 38689658 PMC11059470

# Reviewer report: Reviewer response for version 2

**DOI:** 10.5256/f1000research.161249.r237602

**Published:** 2024-01-25

**Authors:** Stephen Cornish

**Affiliations:** 1 Faculty of Kinesiology and Recreation Management, University of Manitoba, Winnipeg, Manitoba, Canada

Thank you for addressing my previous comments and queries. This is a well written and thought out manuscript.

Is the work clearly and accurately presented and does it cite the current literature?

Yes

If applicable, is the statistical analysis and its interpretation appropriate?

Yes

Are all the source data underlying the results available to ensure full reproducibility?

Yes

Is the study design appropriate and is the work technically sound?

Yes

Are the conclusions drawn adequately supported by the results?

Yes

Are sufficient details of methods and analysis provided to allow replication by others?

No

Reviewer Expertise:

Biomarkers, skeletal muscle, aging, sarcopenia, osteoarthritis

I confirm that I have read this submission and believe that I have an appropriate level of expertise to confirm that it is of an acceptable scientific standard.

# Reviewer report: Reviewer response for version 1

**DOI:** 10.5256/f1000research.144570.r206343

**Published:** 2023-10-17

**Authors:** Amelie M Lutz

**Affiliations:** 1 Radiology, Stanford University, Stanford, USA

This is a well-written manuscript on the an explorative study of load-induced blood marker kinetics in patients with medial knee compartment osteoarthritis are associated with accumulated load and patient-reported outcome measures.

 The authors measured six blood serum biomarkers of cartilage synthesis, cartilage degradation, joint lubrication, or inflammation after a walking test for an extended period up to 5.5 h after the walking test. Of these six blood biomarker, only COMP and MMP-3 showed a consistent increase immediately after the walking test, and COMP and MMP-3 kinetics in response to the mechanostimulus correlated as did load-induced MMP-9 and resistin kinetics.

 The main limitation of the study is the low patient number. The authors list additional limitations.

 Specific comments for the authors :

 In the introduction, please change the listing fot he evaluated blood biomarkers according to their category, meaning list which ones belong to the cartilage synthesis, cartilage degradation groupds and so on. This will make it a much more valuable manuscript also to more general physician readers. 

 Materials and Methods:

 How long was the walking activity? I did not see a defined time period of walking in this section, only briefly mentioned in a subsentence in the discussion (30 minutes?). Was the time duration the same for everybody?

 How were the time intervals for blood draws chosen? Please explain.

 How accurate do the authors think their estimates are of the load accumulated during the walking stress test and assessing the effect of the accumulated load on blood marker kinetics in response to load? Can this really correct for the variability of stress at a self-paced walking exercise?

Is the work clearly and accurately presented and does it cite the current literature?

Yes

If applicable, is the statistical analysis and its interpretation appropriate?

I cannot comment. A qualified statistician is required.

Are all the source data underlying the results available to ensure full reproducibility?

Yes

Is the study design appropriate and is the work technically sound?

Yes

Are the conclusions drawn adequately supported by the results?

Partly

Are sufficient details of methods and analysis provided to allow replication by others?

Partly

Reviewer Expertise:

MSK Radiology, peripheral nerve imaging

I confirm that I have read this submission and believe that I have an appropriate level of expertise to confirm that it is of an acceptable scientific standard, however I have significant reservations, as outlined above.

# Response

**Published:** 2024-01-10

**Authors:** Annegret Mündermann

**Affiliations:** University Hospital Basel, Switzerland

“This is a well-written manuscript on an explorative study of load-induced blood marker kinetics in patients with medial knee compartment osteoarthritis are associated with accumulated load and patient-reported outcome measures. The authors measured six blood serum biomarkers of cartilage synthesis, cartilage degradation, joint lubrication, or inflammation after a walking test for an extended period up to 5.5 h after the walking test. Of these six blood biomarkers, only COMP and MMP-3 showed a consistent increase immediately after the walking test, and COMP and MMP-3 kinetics in response to the mechanostimulus correlated as did load-induced MMP-9 and resistin kinetics.”

 Thank you.

 “The main limitation of the study is the low patient number. The authors list additional limitations.”

 Thank you. We have focused our discussion on limitations that are potentially relevant to the interpretation of our data.

 Specific comments for the authors:

 “In the introduction, please change the listing of the evaluated blood biomarkers according to their category, meaning list which ones belong to the cartilage synthesis, cartilage degradation groups and so on. This will make it a much more valuable manuscript also to more general physician readers.”

 Thank you for this suggestion. We have restructured this section accordingly.

 Materials and Methods:

 “How long was the walking activity? I did not see a defined time period of walking in this section, only briefly mentioned in a subsentence in the discussion (30 minutes?). Was the time duration the same for everybody?”

 Thank you for noting this oversight. The walking stress test include a 30-minute walking activity. We have added this information to the Abstract and Methods section for clarity.

 “How were the time intervals for blood draws chosen? Please explain.”

 The time intervals were chosen based on the literature described and cited in the Introduction section. We have added this information to the Methods section accordingly.

 “How accurate do the authors think their estimates are of the load accumulated during the walking stress test and assessing the effect of the accumulated load on blood marker kinetics in response to load? Can this really correct for the variability of stress at a self-paced walking exercise?”

 In this study, we assessed accumulated load using two different approaches. In approach 1, the maximum, rate of increase and impulse of the vertical ground reaction force during treadmill walking were multiplied by the number of steps taken during the walking stress. In the more advanced approach 2, the maximum and impulse of the knee adduction moment, knee flexion moment, total knee compressive force, medial and lateral compartment compressive force as well as medio-lateral shear force were calculated from musculoskeletal modeling for the affected knee during overground walking multiplied by the number of steps taken during the walking stress.

 The number of steps taken during the walking stress and hence the number of load cycles was counted and hence are an accurate measure. Ideally, one would measure the load directly at the tissue level, but this is not possible in vivo in humans for obvious reasons. Any musculoskeletal model used to estimate joint level load is based on some assumptions. In our models, we scaled the generic model to patient-specific anthropometrics and adjusted the varus/valgus alignment estimated from the marker position as described in the cited articles. The accuracy of these models can be further improved by increasing the personalization of the models (i.e., person-specific morphology taken from medical imaging such as magnetic resonance imaging vs. scaled based on anthropometrics) and estimating compartment-specific tissue level pressures. The latter was beyond the scope of this study but will be considered in future projects. We have added this potential limitation to the Limitations section. The reviewer is correct that there may be some variability in tissue level stress during a self-paced walking exercise within an individual. In this study, we measured blood marker kinetics after the walking stress and not during the stress, and we were primarily interested in the variability in blood marker kinetics in response to the walking stress and the variability in accumulated load between participants, which we found to be related. Overall, while the accuracy of the estimates depends on the underlying assumptions, but the observed correlations of blood marker kinetics and accumulated load supports our hypothesis.

 “Is the work clearly and accurately presented and does it cite the current literature? – Yes”

 Thank you.

 “Is the study design appropriate and is the work technically sound? – Yes”

 Thank you.

 “Are sufficient details of methods and analysis provided to allow replication by others? – Partly”

 We have added information on the methods and analysis as requested that is important for allowing replication by others.

 “If applicable, is the statistical analysis and its interpretation appropriate? – I cannot comment. A qualified statistician is required.”

 All statistical analyses were performed by an experienced biostatistician.

 “Are all the source data underlying the results available to ensure full reproducibility? – Yes”

 Thank you.

 “Are the conclusions drawn adequately supported by the results? – Partly”

 We hope that with the revisions made, the reviewer agrees that our conclusions are supported by the results.

# Reviewer report: Reviewer response for version 1

**DOI:** 10.5256/f1000research.144570.r195180

**Published:** 2023-09-08

**Authors:** Stephen Cornish

**Affiliations:** 1 Faculty of Kinesiology and Recreation Management, University of Manitoba, Winnipeg, Manitoba, Canada

This research study evaluated the correlations between a number of blood based biomarkers and patient reported outcomes as well as accumulated loads during a 30-minute walking protocol in patients with knee OA. The number of biomarkers assessed is a strength in the study design. I only have a few comments for the authors to consider in making revisions to the manuscript:

1. I realize this was an exploratory study; however, it would be appropriate to include a hypothesis statement in the introduction.
2. In the methodology it is mentioned that the patients were asked to refrain from physical activity for 24 hours prior to testing. I believe this would be nearly impossible, so should be rephrased or eliminated from the methodology.
3. Figure 1 is very difficult to see in its present form. Could this be enlarged to show where the significant differences are located more clearly in the 11 biomarkers?
4. In the methodology a more thorough explanation of how the ELISAs were completed should be included.
5. Minor comment - sentence 2 of the conclusion should read:"... CPII/C2C, IL-6..." not 'C3C' I believe.

Overall, this is a well written manuscript with some interesting and novel data.

Is the work clearly and accurately presented and does it cite the current literature?

Yes

If applicable, is the statistical analysis and its interpretation appropriate?

Yes

Are all the source data underlying the results available to ensure full reproducibility?

Yes

Is the study design appropriate and is the work technically sound?

Yes

Are the conclusions drawn adequately supported by the results?

Yes

Are sufficient details of methods and analysis provided to allow replication by others?

No

Reviewer Expertise:

Biomarkers, skeletal muscle, aging, sarcopenia, osteoarthritis

I confirm that I have read this submission and believe that I have an appropriate level of expertise to confirm that it is of an acceptable scientific standard, however I have significant reservations, as outlined above.

# Response

**Published:** 2024-01-10

**Authors:** Annegret Mündermann

**Affiliations:** University Hospital Basel, Switzerland

“This research study evaluated the correlations between a number of blood based biomarkers and patient reported outcomes as well as accumulated loads during a 30-minute walking protocol in patients with knee OA. The number of biomarkers assessed is a strength in the study design. I only have a few comments for the authors to consider in making revisions to the manuscript.”

 We have carefully considered your comments and hope to have addressed these to your satisfaction.

 “I realize this was an exploratory study; however, it would be appropriate to include a hypothesis statement in the introduction.”

 We rephrased our study aims to include hypotheses that are in line with the published study protocol (reference 33). Specifically, we tested the hypotheses that (i) some but not all blood markers will respond to the walking stress, and that the kinetics in response to a walking stress correlates (ii) between some but not all markers, (iii) with accumulated knee load during the walking stress, and (iv) with patient reported osteoarthritis outcome and QoL.

 “In the methodology it is mentioned that the patients were asked to refrain from physical activity for 24 hours prior to testing. I believe this would be nearly impossible, so should be rephrased or eliminated from the methodology.”

 It is correct that patients were not asked to refrain from physical activity as this would be nearly impossible. We have corrected this statement to the actual instructions given, which were to minimize their physical activity for 24 hours prior to testing.

 “Figure 1 is very difficult to see in its present form. Could this be enlarged to show where the significant differences are located more clearly in the 11 biomarkers?”

 We agree that the figure is compact in its printed form. To enlarge the individual graphs, the figure would have to be split into three separate figures which would have the disadvantage of losing the difference in mechanoresponse to the walking stress between the different blood markers. Since the figure can be enlarged as desired in the online and pdf versions, we prefer to leave the figure as it is.

 “In the methodology a more thorough explanation of how the ELISAs were completed should be included.”

 All ELISAs were performed according to the manufacturers’ instructions. We have included this information in the Methods section. All manufacturers are listed in Table 2, and the instructions are available on the manufacturers’ websites. Therefore, we do not believe that describing the ELISA for each blood marker in detail is necessary or indicated. However, if the reviewer or the editor would prefer to include this information in the article, we would be happy to do so.

 “Minor comment - sentence 2 of the conclusion should read:"... CPII/C2C, IL-6..." not 'C3C' I believe.”

 Thank you for noting this oversight. We have corrected this typo.

 “Overall, this is a well written manuscript with some interesting and novel data.”

 Thank you.

 “Is the work clearly and accurately presented and does it cite the current literature? – Yes”

 Thank you.

 “Is the study design appropriate and is the work technically sound? – Yes”

 Thank you.

 “Are sufficient details of methods and analysis provided to allow replication by others? – No”

 We assume that this comment referred to the information on the pre-stress test activity, the duration of the stress test and the ELISAs. We have included this information as requested.

 “If applicable, is the statistical analysis and its interpretation appropriate? – Yes”

 Thank you.

 “Are all the source data underlying the results available to ensure full reproducibility? – Yes”

 Thank you.

 “Are the conclusions drawn adequately supported by the results? – Yes”

 Thank you.
